# Investigation of Spectroscopic Peculiarities of Ergot-Infected Winter Wheat Grains

**DOI:** 10.3390/foods12183426

**Published:** 2023-09-14

**Authors:** Dmitrii Pankin, Anastasia Povolotckaia, Eugene Borisov, Alexey Povolotskiy, Sergey Borzenko, Anatoly Gulyaev, Stanislav Gerasimenko, Alexey Dorochov, Viktor Khamuev, Maksim Moskovskiy

**Affiliations:** 1 Center for Optical and Laser Materials Research, St. Petersburg State University, Ulianovskaya 5, 198504 St. Petersburg, Russia; dmitrii.pankin@spbu.ru (D.P.); eugene.borisov@spbu.ru (E.B.); 2 Institute of Chemistry, St. Petersburg State University, Universitetskii pr. 26, 198504 St. Petersburg, Russia; alexey.povolotskiy@spbu.ru; 3 Federal Scientific Agro-Engineering Center VIM, 1st Institutskiy proezd 5, 109428 Moscow, Russia; serzhbk@gmail.com (S.B.); tomasss1086@mail.ru (A.G.); stanislav.mkm@gmail.com (S.G.); dorokhov@rgau-msha.ru (A.D.); viktor250476@yandex.ru (V.K.); maxmoskovsky74@yandex.ru (M.M.)

**Keywords:** wheat, ergot, Raman spectroscopy, FTIR, UV-vis-NIR, luminescence

## Abstract

Wheat has played an important role in human agriculture since ancient times. Increasing rates of processed wheat product fabrication require more and more laboratory studies of product quality. This, in turn, requires the use, in production and in field conditions, of sufficiently accurate, fast and relatively low-cost quality control methods, including the detection of fungal diseases. One of the most widespread fungal diseases of wheat in the world is ergot caused by the fungi genus *Claviceps*. Optical methods are promising for this disease identification due to the relative ease of implementation and the possibility of performing fast analyses in large volumes. However, for application in practice, it is necessary to identify and substantiate characteristic spectral markers that make it possible to judge the sample contamination. In this regard, within the framework of this study, the methods of IR absorption spectroscopy in the MIR region and reflection spectroscopy in the UV-vis-NIR ranges, as well as luminescence spectroscopy, were used to study ergot-infected grains of winter wheat of the “Moskovskaya 56” cultivar. To justify the choice of the most specific spectral ranges, the methods of chemometric analysis with supervised classification, namely PCA-LDA and PCA-SVM, were applied. The possibility of separating infected grains according to the IR absorption, reflection spectra in the UV-vis-NIR ranges and visible luminescence spectra was tested.

## 1. Introduction

Wheat has played an important role in agriculture since ancient times, being a widespread and valuable source of nutrients [1,2]. However, the susceptibility of wheat varieties to infection with fungal diseases can lead to a significant loss of yield and loss of livestock, as well as a threat to the life and health of humans and animals [1,3,4,5].

Among such fungal infections is the widespread ergot caused by infection with genus fungi *Claviceps* sp. Among these fungi, one of the most common is *Claviceps purpurea Tul* (Fr.) growing on rye, wheat, barley and other oats [6]. Ergot alkaloids have a toxic effect on human and animal organisms, leading to the development of ergotism (food toxicosis) when consumed in significant quantities [5,6]. In this regard, in a number of countries, maximum allowable concentrations for ergot alkaloids or ergot bodies, as well as internal and interstate grain quality standards and recommendations are established [1].

Up-to-date studies summarize the approaches directed at lowering the level of ergot disease [3], in particular agricultural practices, applications of current and perspective fungicides [7] and chemical inhibitors, as well as genetical resistance improvement. The timely identification and quality control of both seed material and stored grains intended for further consumption by animals and humans are essential in preventing the spread of ergot.

To date, the main methods for identifying ergot fungi and their metabolites are visual grain examination, chromatographic methods (thin-layer chromatography, liquid chromatography with mass spectrometry, high-performance liquid chromatography (HPLC) with UV-vis absorbance), enzyme-linked immunosorbent assay (ELISA) and methods of optical spectroscopy (absorption spectroscopy in the near-IR (NIR) region, IR absorption spectroscopy in the middle-IR (MIR) region, Raman and surface-enhanced Raman scattering (SERS) spectroscopy, infrared hyperspectral imaging) [4,8,9,10,11,12,13]. The limitations associated with visual grain examination are based on the need to characterize large sample amounts. Specimens must grow to a size sufficient for morphological identification. The qualifications of the specialist making the decision are also essential. Chromatographic methods suggest high precision. But their disadvantages include the complexity of equipment operation, the need for pure reagents, and these methods being time-consuming. To conduct and interpret data obtained by chromatographic and ELISA methods, highly qualified specialists are required. All this makes it difficult to use these methods for streaming analysis with a large number of samples.

At the same time, optical spectroscopy methods have a number of advantages associated with a sufficiently high sensitivity to the structure of the studied substances, the possibility for resonant identification of biodamage by specific pigments (their presence or absence), the possibility of predicting the optical properties of fungal metabolites using density functional theory (DFT) methods and the relative simplicity and low cost of implementing devices based on practice, as well as the relatively quick analysis [14,15,16,17,18,19]. Moreover, the application of chemometric techniques allows one to identify implicit dependencies, reduce the data dimensions and highlight the higher-impact spectral regions.

For example, the NIR hyperspectral imaging method with partial least-squares discriminant analysis (PLS-DA) was used to detect particles of ergot bodies online [20]. A number of articles were devoted to the study of ergot alkaloids in wheat and barley using a combination of Fourier transform infrared (FTIR) and NIR spectroscopy methods combined with chemometric analysis [21,22]. FTIR and NIR spectroscopy are promising for studying the possibility of detecting fungal infection [23]. The presence of characteristic IR absorption peaks for individual functional groups in the fingerprints region of the MIR spectra allows one to analyze the changes occurring with substances at the molecular level and the level of individual functional groups. Recently, a number of studies [24,25] demonstrated the promise of studying the UV-vis spectral features that allow one to separate infected and healthy grains during fungi infection.

In this regard, within the framework of this research, the FTIR absorbance in the MIR regions, UV-vis-NIR reflectance and luminescence spectroscopy in the UV-vis regions techniques were chosen. This work is aimed at the spectral features determination for the reliable differentiation of infected and healthy grains with the abovementioned techniques. This combination of techniques contributes to a more reliable infection identification and allows one to use the complementary method in conditions where one of the methods is insensitive.

The substantiation of the selected spectral ranges for the differentiation of healthy and infected grains, as well as the testing of the predictive ability, was carried out using linear discriminant analysis (LDA) and the support vector machine (SVM) technique on the basis of data (scores) in new coordinates (several first principal component vectors) corresponding to every spectrum obtained with principal component analysis (PCA). They are the so-called PCA-LDA and PCA-SVM approaches. The choice of these chemometric methods was made on the basis of their earlier successful application for detection of *Fusarium sp*.-infected “Zalp” cultivar oat grains [26].

Further use of certain spectral features may be applicable for the differentiation of ergot-infected and healthy wheat grains on an industrial scale, as well as subsequent implementation in streaming control systems.

## 2. Materials and Methods

### 2.1. Plant Materials

The grains of winter wheat (*Triticum aestivum* L.) of the “Moskovskaya 56” variety were selected for the investigation. The variety was bred by crossing (Mironovskaya semi-intensive × Inna) × Moskovskaya 39, and it has appeared in registers of seed farms since 2008 [25]. The investigated samples were grown in the Central Black Earth region of Russia the subzone 1.1 and 1.2 (agricultural fields of The Federal Scientific Agro-Engineering Center VIM at coordinates 54°34′56.5″ N 39°32′42.0″ E) in the 2021 year. The weight determined for 1000 grains is in the range of 39–45 g [26]. The average grain weight of investigated healthy samples selected for the research was about 41 mg. The advantages of this variety are increased productivity, winter hardiness, resistance to lodging, leaf rust and powdery mildew. Moreover, this variety is valued for its good baking qualities. The details concerning the infected grains are described in Section 2.2.

### 2.2. Grain Selection

The total weight of the sample was 3.2 kg. Identification of infection was carried out at the Federal Center for Assessing the Safety and Quality of Grain and Their Processing Products (FGBU “Center for Assessing Grain Quality”) by the Federal Service for Veterinary and Phytosanitary Supervision (Rosselkhoznadzor of the Russian Federation). The interstate standard for infection confirmation: “GOST 12044-93 Agricultural grains. Methods for determination of disease infestation”, was used. The presence of 78% of infected grains was found. These grains have significant visual differences. Infected grains were black with a purple tint and unequal banana-shaped, and significantly exceeded the average size and weight of healthy grains. Thus, the average weight of infected grains was 182 mg, which is 4.4 times higher than the average weight of healthy grains. The 39 healthy and 39 infected grains were selected for FTIR absorbance and luminescent spectral studies due to the spectrometer accessory mounting system features. A total of 21 healthy and 21 infected grains were selected for UV-vis-NIR reflectance spectral study. The grains slightly varied in size and shape, making some of them difficult to mount on the accessory. The spectra acquisition in all cases was performed from a grain with no mechanical impact. All the healthy and infected grains were examined and the grains classification was confirmed according to techniques described above.

### 2.3. FTIR Spectroscopy

IR absorption spectra in the MIR region were measured by attenuated total reflection (ATR) technique using the Nicolet 8700 FTIR spectrometer (Thermo Fisher Scientific; Waltham, MA, USA) (hereinafter FTIR spectra). The diameter of diamond ATR crystal was about 2 mm. To obtain the spectra, the MCT-A detector with liquid nitrogen cooling was selected. Spectra were obtained under the following conditions: 4 cm^−1^ spectral resolution; 150 scans; the phase correction was performed according to the Mertz method [27,28]; and the Blackman–Harris window function [29,30] was chosen as the apodization function. The spectra were obtained in the range 650–4000 cm^−1^ from the grain as it is. The obtained spectra were normalized in the range (0, 1); 39 spectra of healthy and 39 spectra of infected grains were randomly selected to demonstrate the chemometric analysis application. Much less informative regions in the range 650–715 cm^−1^ and 1900–2700 cm^−1^ were omitted. In both cases, the averaged spectra were obtained by averaging over 39 spectra of each sample type. The difference spectrum was calculated as averaged spectrum for infected grains minus averaged spectrum for healthy grains. Spectra processing was performed in the OriginPro2021b (OriginLab Co.; Northampton, MA, USA).

### 2.4. UV-vis-NIR Reflectance Spectroscopy

Reflection spectra were obtained on a Lambda 1050 precision spectrophotometer (Perkin Elmer, Waltham, MA, USA) in the range 200–2500 nm (UV-vis-NIR) using integration sphere accessory, 150 mm in size. The scanning step was 4 nm, the scanning speed was 2 nm/s. Two detectors were used in order to cover the whole range 200–2500 nm, namely the R6872 PMT detector for 200–860 nm and InGaAs one for 860–2500 nm range. Spectralon White was selected as the reference sample. Before the measurements, calibration was carried out at 100% and 0% reflection. A total of 21 reflectance spectra for healthy grains and 21 reflectance spectra for infected grains were obtained. The spectra were obtained from the grain as it is. The averaged spectra for healthy and infected cases were obtained in the OriginPro2021b (OriginLab Co.; Northampton, MA, USA).

### 2.5. Luminescence Spectroscopy

Luminescence excitation and emission spectra were carried out using a modular fluorimeter Fluorolog-3 (Horiba Jobin Yvon, Longjumeau, France). The scanning step was 1 nm. The excitation source was 450 W Xe lamp, the detector was CCD matrix. The luminescence emission spectra of infected and healthy grains were obtained in the ranges of 350–650 and 600–1000 nm with excitation at 336 and 400 nm, respectively. Additional excitation spectra were collected in the range 290–430 nm and 260–650 nm for healthy grains with luminescence maxima at 450 and 670 nm. The spectra were collected from 39 infected and 39 healthy samples. The spectra were obtained from the grain as it is. The averaged spectra for healthy and infected cases were obtained in the OriginPro2021b (OriginLab Co.; Northampton, MA, USA). A total of 39 infected and 39 healthy grain emission spectra in the 600–1000 nm range with 400 nm excitation were normalized by maximum via OriginPro2021b (OriginLab Co.; Northampton, MA, USA).

### 2.6. Data Analysis

#### 2.6.1. Chemometric Analysis

Due to the large variance and complex spectral multidimensional behavior in MIR absorbance spectra and in UV-vis-NIR reflectance spectra, it was decided to apply chemometric techniques. Such analysis made it possible to reduce data dimensions as soon as the highly impact spectral regions were identified. The analysis was performed within two stages [31]. At the first stage, a linear transition to the new orthogonal variables the so-called principal components (PCs) was carried out, and the scores were obtained for corresponding spectra. The regions with the highest weights in loading spectra, which have the greatest influence, were identified. The ordering of the main components was carried out in order of decreasing variability, which allowed one to reduce the dimensions of the spectral data to several components that accumulate a fairly large amount of information. In this paper, it was decided to take into consideration the first three components that accumulate >90% of information. In this article, the normalized FTIR, UV-vis-NIR and luminescence spectra (see their processing descriptions in Section 2.3, Section 2.4 and Section 2.5) were analyzed by the principal component analysis method in the OriginPro2021b software (OriginLab Co.; Northampton, MA, USA) and, in particular, Principal Component Analysis for Spectroscopy v1.3 application incorporated in it.

At the next stage, the scores obtained for the first three principal components in different combinations were used for supervised machine learning and the predictive model testing. The classification method based on the SVM technique was chosen as an approach. In such an approach, the hyperplane in k-space is derived during the training at the corresponding set in order to construct classification conditions [32]. To demonstrate the application in solving nonlinear problems, the radial basis function (RBF) (Gaussian type) kernel [33] was chosen, which showed better results in comparison with various polynomial kernels [26,33]. Also, the widely used LDA technique was chosen for comparison the classification results. With the same data sets all calculations were performed using OriginPro2021b (OriginLab Co.; Northampton, MA, USA), and in particular, SVM Classification software v1.7 was incorporated in it. Spectra from 26 healthy and 26 infected grains were arbitrarily chosen as a training set in the case of IR absorption spectra in the MIR region, as well as spectra from 13 healthy and 13 infected grains, in order to test the predictive power: 39 spectra of each of two classes. In total, there were 78 items. These spectra are discussed in Section 2.3. In the same way, spectra from 26 healthy and 26 infected grains were arbitrarily chosen as a training set in the case of luminescence spectra for the 600–1000 nm region with 400 nm excitation, as well as spectra from 13 healthy and 13 infected grains in order to test the predictive power: 39 spectra of each of two classes. In total, there were 78 items. These spectra are discussed in Section 2.5.

In the case of UV-vis-NIR reflectance spectra for training sets, spectra from 14 healthy and 14 infected grains were randomly selected, as well as spectra from 7 healthy and 7 infected grains in order to test predictive power: 21 spectra of each of two classes. In total, there were 42 items; these spectra are discussed in Section 2.4.

#### 2.6.2. Model Evaluation

The predictive power estimation for discrimination against infected grains (positive result) and healthy grains (negative result) using the above approaches was carried out based on such commonly used criteria as sensitivity, specificity, positive predictive value, negative predictive value and accuracy, which were defined as follows [31,34,35,36]:

Sensitivity = T_P_/(T_P_ + F_N_),

Specificity = T_N_/(T_N_ + F_P_),

Positive predictive value = T_P_/(T_P_ + F_P_),

Negative predictive value = T_N_/(T_N_ + F_N_),

Accuracy = N_corr_/N_tot_.

where T_P_ is the number of true positive results, T_N_ is the number of true negative results, F_P_ is the number of false positive results, F_N_ is the number of false negative results, N_corr_ is the number of correctly classified samples, N_tot_ is the total number of samples.

## 3. Results and Discussion

### 3.1. MIR Absorption Spectroscopy

#### 3.1.1. FTIR Spectral Trends and Features

The IR absorption spectra in the MIR region obtained by the ATR method have a characteristic depth from which information is obtained. Therefore, as well as the complex structure of the wheat grain, it is necessary to clarify that the spectra obtained by this method carry information about the outer layers.

The outermost layer of the grain is its husk. According to [37], wheat husk is mainly composed of predominantly cellulose and hemicellulose, with a smaller percentage of lignin and starch. The next layer types are the outer and inner pericarp, which consist mainly of cellulose, lignin and heteroxylans that form empty cells [36,38].

**Figure 1:**
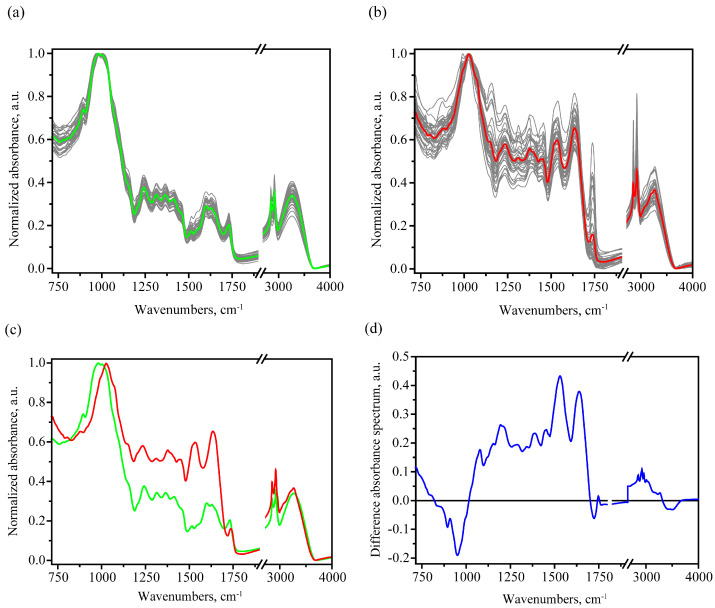
FTIR absorbance spectra from 39 healthy (**a**) with the corresponding averaged spectrum (green) and 39 ergot-diseased (**b**) wheat grains with the corresponding averaged spectrum (red); comparison of the averaged and normalized absorbance spectra of healthy (green) and infected (red) cases (**c**) and the difference absorbance spectrum obtained as the averaged infected absorbance spectrum minus the averaged healthy absorbance spectrum (**d**).

This composition correlates with the observed features in the obtained FTIR absorption spectra for healthy grains (see Figure 1a). The observed main peaks and their interpretation based on [26,39,40,41,42,43] are given in Table 1.

After normalization, a characteristic feature of the obtained FTIR absorption spectra from healthy seed samples is a predominant signal in the carbohydrates region, with much lower absorption in the 1200–1900 cm^−1^ region, not exceeding 0.44 a.u. for the averaged spectrum in the healthy case. A much higher relative absorption is noted for infected samples in this range, which is clearly seen when comparing individual spectra in Figure 1a,b, as well as during the averaged spectra comparison in Figure 1c. In the latter, the average infected spectrum is characterized by the presence of pronounced protein peaks: amide I (1633 cm^−1^), amide II (1534 cm^−1^) and amide III (1233 cm^−1^) (see Figure 1 and Table 2). In addition, an increase in the carbohydrate absorption band maximum frequency was noticed for the infected case. In the case of the healthy sample averaged spectrum, the carbohydrate peak is broad near the maximum (974–1009 cm^−1^), and the exact maximum is situated at about 983 cm^−1^. On the other hand, in the case of infected sample averaged spectrum, the carbohydrate peak is narrower, and the maximum is about 1027 cm^−1^ (see Figure 1c).

During the comparative analysis of FTIR spectra from infected and uninfected areas, one can also note a much larger dispersion in the absorption values for spectra from infected areas, which is associated with their greater degree of defectiveness, lower uniformity and qualitatively different composition.

#### 3.1.2. PCA of FTIR Absorption Spectra

The PCA method was applied in order to make a more reliable selection of the spectral feature characteristic for the infected sample. This analysis was carried out in the spectral region consisting of the ranges 715–1900 cm^−1^ and 2700–4000 cm^−1^. The obtained values of the cumulative percent of variance, depending on the number of the component, are shown in Figure S1. In this paper, it was decided to limit to the first three principal components, which account for just over 96% of the data. Moreover, the first component makes it possible to explain 88.3%.

**Figure 2:**
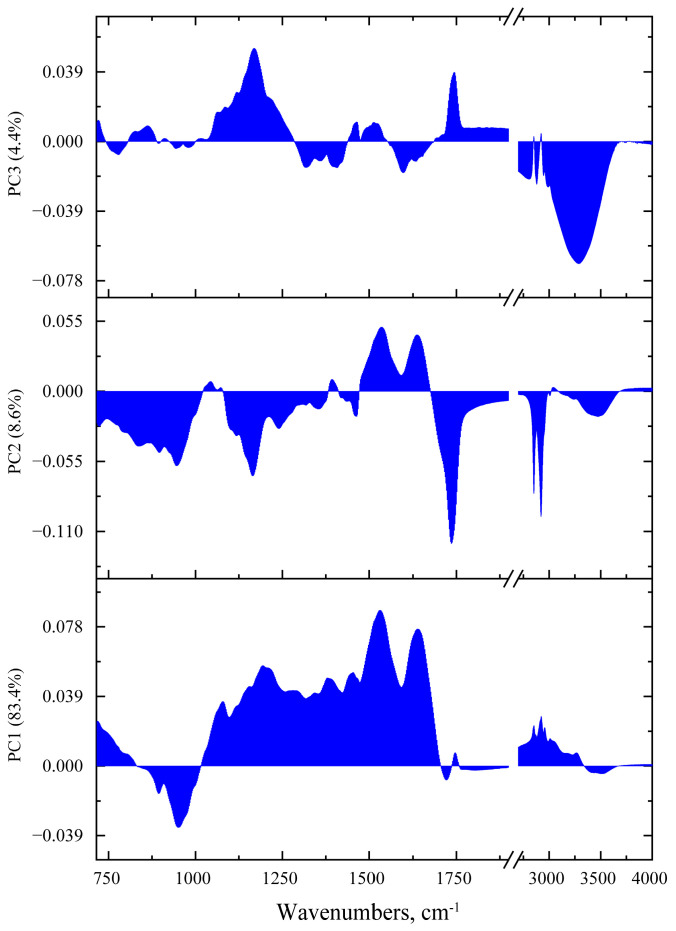
The loadings of first 3 PCs obtained during PCA analysis of FTIR absorbance data: bottom for PC1, in the middle for PC2 and at the top for PC3.

Loadings for the first three PCs are shown in Figure 2. Considering the PC1 case, the loading curve correlates with the difference spectrum shown in Figure 1d. The largest loading weights for the first component are in the spectral range 1130–1690 cm^−1^, while the maximum values are close to the position of the amide peaks, namely 1203, 1532 and 1639 cm^−1^. Thus, the loading curve of the first component, selected according to the principle of the greatest data variability, has similarities with the characteristic IR absorption spectra of protein media that may reflect the protein contribution of fungi to the spectra.

The PC2 loading spectrum is characterized by a greater degree of localization of the largest weights compared to PC1. The largest weights for PC2 are present in the region characteristic for C-H stretching vibrations in methylene groups (2851 and 2922 cm^−1^), in the region of stretching C=O bond vibrations (1736 cm^−1^), as well as for antisymmetric stretching vibrations in single carbon–oxygen bonds, including in ether and ester groups (about 1164 cm^−1^) and carbohydrates (range 870–960 cm^−1^); the interpretation is based on [40,44]. The weights in the region of the amide I and amide II peaks anticorrelate with the previous. In general, it can be noted that the nature of the loading for PC2 in the region of more than 1050 cm^−1^ correlates with typical lipid spectra. This may be due to the fact that in grains infected with ergot genus fungi, a complex mixture of stable lipids is produced as metabolic products [45].

The loading for the PC3 is characterized by the largest weights in the region of OH stretching vibrations; the maximum weight is reached at about 3287 cm^−1^. This may be a manifestation in the different moisture content between healthy and infected grains. The peaks at 1167 and 1742 cm^−1^ anticorrelate with the OH stretching vibration peak.

**Figure 3:**
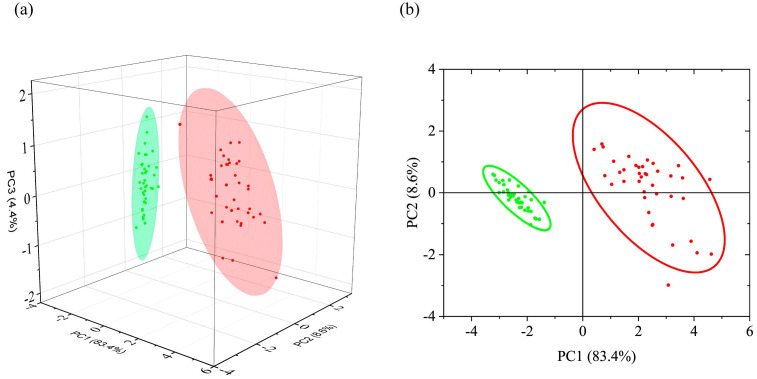
Combination of scores of principal components: (**a**)—(PC1, PC2, PC3); (**b**)—(PC1, PC2) for healthy (green) and infected (red) samples with corresponding 95% probability ellipsoids.

The scores obtained for the first three principal components, and the classifications using their various pair combinations are shown in Figure 3 and Figure S2. The Euclidean distance between the centers of the 95% probability ellipsoids are 4.73, 4.73, 4.71, 0.38, respectively (the values rounded to the second digit). A distinct separation is observed when the first principal component is used in the combination of scores. The scores for healthy and infected samples anticorrelate over it. The Euclidean distance between the centers of the 95% probability ellipsoids are close in case of combinations with PC1.

#### 3.1.3. LDA and SVM Classification

The corresponding estimations of such parameters as accuracy, sensitivity and specificity, positive predictive value and negative predictive value for the PCA-LDA and PCA-SVM classification models at the same time reach the maximum values for both the training and test data sets (Table 3). However, for the case when scores for PC2 and PC3 were used, there is no explicit separation; the scores for healthy samples are inside the area for infected ones. However, it should be noted that the data localization area for healthy samples is significantly smaller, primarily due to PC2. This circumstance, as well as the use of a nonlinear RBF kernel when finding hyperplanes in the SVM method, made it possible to perform separation with greater accuracy using the PCA-SVM method than PCA-LDA (Table 3). In the cases of scores for PC2 and PC3, the Euclidean distance between the ellipsoids of 95% probability was the smallest of the four cases presented.

The estimated accuracy parameters of the models demonstrate the greater predictive accuracy for the PCA-SVM approach, adapted to solving nonlinear tasks, compared to PCA-LDA (see Table 3). Qualitatively similar results for predictive ability were noted in the work [31].

Summing up, for this stage of infection, the fundamental possibility of separating on the basis of IR absorption spectra into healthy and infected grains using the PCA-LDA and PCA-SVM approaches was demonstrated, and the maximum efficiency was achieved for a combination of scores with PC1.

### 3.2. UV-Vis-NIR Spectroscopy

#### 3.2.1. UV-Vis-NIR Spectral Trends and Features

**Figure 4:**
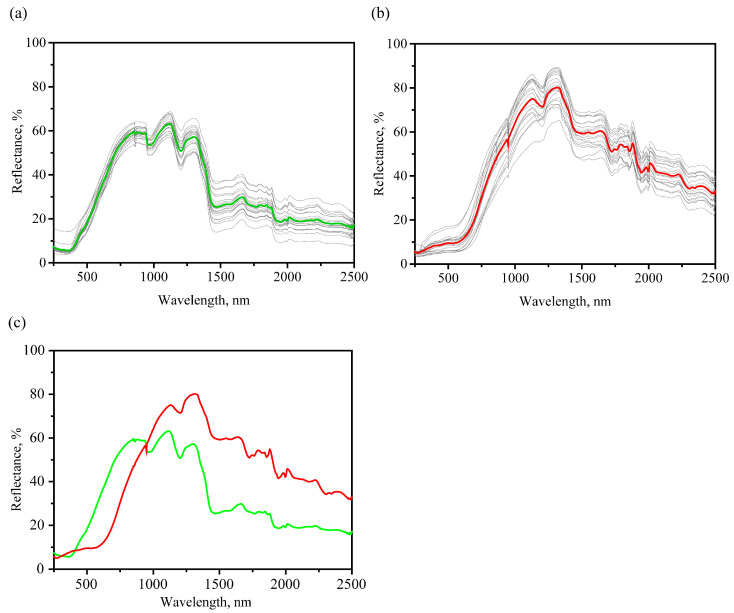
Comparison of the UV-vis-NIR absorbance spectra of healthy samples with the corresponding averaged spectrum (green) (**a**) and infected samples spectra samples with the corresponding averaged spectrum (red) (**b**); comparison of the averaged UV-vis-NIR reflectance spectra of healthy (green) and infected (red) cases (**c**).

A number of characteristic spectral features can be distinguished in the obtained reflection spectra; see Figure 4. When comparing the averaged spectra of healthy grains (see Figure 4c), a flat reflection minimum was observed in the range 265–369 nm. At the same time, a clearly observed minimum near 269 nm and a longer wavelength shoulder at approximately 543 nm were observed for the infected grains. In the region from 400 to 960 nm for the case of infected grains, a much smaller reflection is noted than in the case of healthy grains. This coincides with the visual perception of infected grains as darker ones for the part of the spectrum corresponding to the visible region. Possible differences in absorption spectra in these regions are related to the presence of red anthraquinone pigments (e.g., clavorubin, endocrocin) as well as yellow ergochrome pigments [46].

A number of absorption bands are noted on the averaged spectra in the region above 1000 nm. The most significant bands correspond to the following minimums in the reflectance spectra (interpretation made in accordance with [47,48,49]): 1200 nm (second overtone of C-H stretching vibrations), 1468 nm (broad, first overtone of O-H and N-H stretching vibrations), 1720 nm (first overtone of C-H stretching vibrations), 1940 nm (combination including O-H stretching vibrations). On average, the percentage of reflection for the case of infected grains is higher than for healthy ones in the region more than 1000 nm.

In general, the difference in the averaged UV-vis-NIR reflectance spectra is associated with the following reasons: a significant change in the chemical composition (e.g., appearance of fungi pigments, alkaloids and other fungi metabolites), as well as a transition from more structured ordered materials (such polymers as cellulose or hemicellulose in the outer layers in healthy grains) to less ordered (more amorphous substances).

#### 3.2.2. PCA of UV-vis-NIR Absorption Spectra

Chemometric techniques were used to study the possibility of separating grains into healthy and infected on the basis of UV-vis-NIR reflectance spectra. At the initial stage, a transition to the dimensionally reduced data was made by the PCA. The obtained values of the eigenvalues, as well as the cumulative percent of variance, depending on the number of the component, are shown in Figure S3. Together, the three first PCs represent about 99% of the variance. In such a way was chosen the score set of the three first PCs for the purposes of further classification possibility tests.

**Figure 5:**
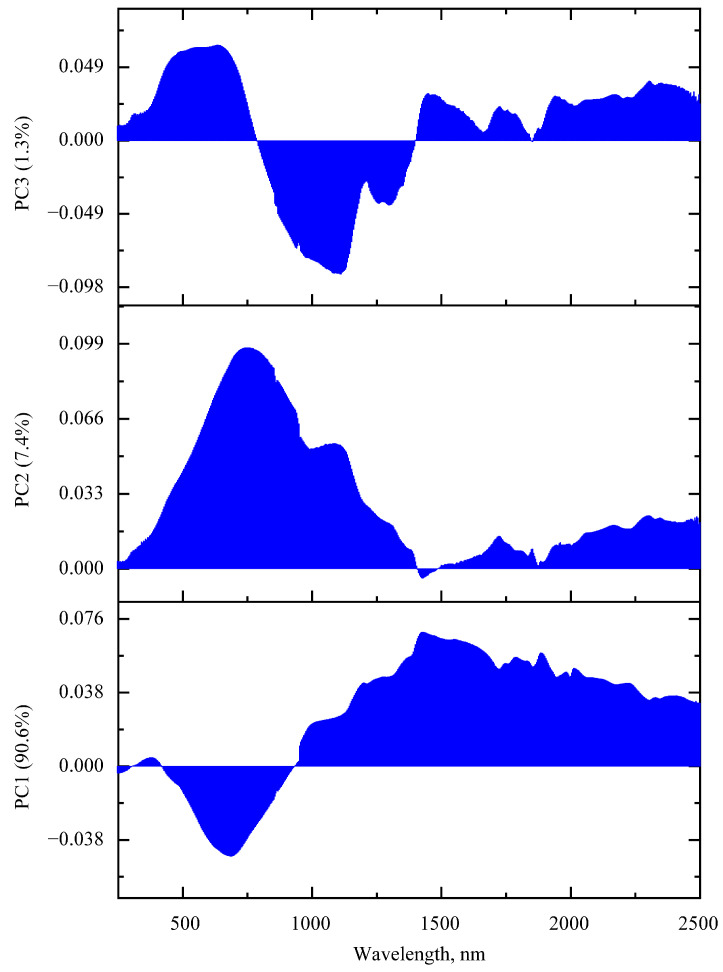
The first three PCs loading spectra obtained during PCA analysis of UV-vis-NIR reflectance data: bottom for PC1, in the middle for PC2 and at the top for PC3.

Loading graphs are shown in Figure 5. Considering PC1, the largest weights on the loading graph are present in the region of 1000–2500 nm, i.e., in the region where overtones for stretching vibrations in O-H, C-H, N-H bonds and their combinations appear. With the largest weight at 683 nm, the red spectral region anticorrelates with the previous region. PC1 alone explains about 90.6% of all spectra. For PC2, the largest weights are observed at 750 nm, and in contrast to PC1, the largest loadings are already localized in the region 445–1180 nm, i.e., a significant contribution is made in the visible and near-IR ranges. The total explained percentage for the first two components is 98%. As for PC3, there is a much greater degree of localization. In the PC3 loading spectrum, the greater weights are in the ranges 455–700 nm and 940–1150 nm.

#### 3.2.3. LDA and SVM Classification

**Figure 6:**
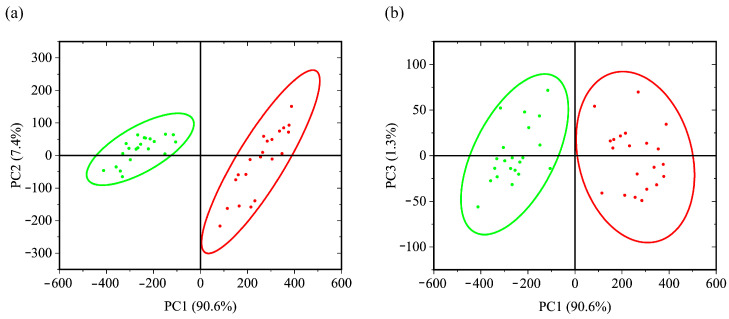
Combination of scores of principal components: (**a**)—(PC1, PC2), (**b**)—(PC1, PC3) for healthy (green) and infected (red) samples with corresponding 95% probability ellipsoids.

The results of classification with the PCA-LDA and PCA-SVM approaches are shown in Figure 6 and Figure S4, as well as Table 4. It can be seen that the score localization occurs in two- and three-dimensional space mainly due to PC1. The Euclidean distance between the projections of the 95% probability ellipsoid centers on the two-dimensional subspace for healthy and infected samples decreases in the series (PC1,PC2) 516 a.u. > (PC1, PC3) 515 a.u. > (PC2, PC3) 42 a.u. The 95% probability ellipsoids of infected and uninfected grains overlap for the combination of PC2, PC3, which leads to much lower predictive accuracy. The situation with localization of scores in the PC2, PC3 space is similar to the case described in Section 3.1.2. And in this situation, the PCA-SVM method also predicts with greater accuracy than PCA-LDA. At the same time, expanding the data set to the first three principal components still leads to an error-free classification. The results of tests are the same as in the case of the sets (PC1, PC2) and (PC1, PC3). Based on all of the aforementioned, the classification made using the first two components could be considered as the simplest and most effective.

### 3.3. Luminescence Spectroscopy

#### 3.3.1. Luminescent Spectral Trends and Features

Luminescent spectroscopy is a sensitive technique to study the substance composition, and is closely related to its electronic structure and transition probabilities. Currently, the luminescent properties of ergot metabolites, such as alkaloids, are used in conjunction with HPLC to confirm infection. It should be noted that in a number of studies [8,50,51] aimed at studying ergot alkaloids, the luminescence control of their presence consists of the 420 nm wavelength luminescence detection under 250 nm excitation. By itself, such a technique is labor-intensive and time-consuming, as well as requiring the extraction of necessary substances from infected grains. Therefore, the search for luminescent features that allow one, without interfering with the structure of the seed, to distinguish healthy grains from infected ones is of great interest. The use of widespread optical components for portable luminescent spectrometers designed for UVA-vis-NIR regions is especially promising for such measurements. In this regard, the luminescence features of healthy and ergot-infected grains were also studied in the range of 350–1000 nm.

**Figure 7:**
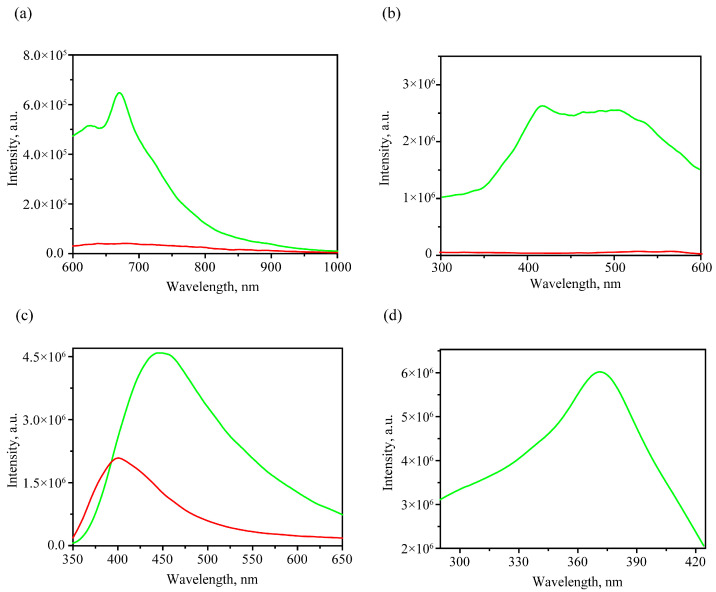
Averaged emission spectra in 600–1000 nm with 400 nm excitation (**a**) and averaged excitation spectra for 670 nm peak (**b**); averaged emission spectra in 350–650 nm with 336 nm excitation (**c**) of healthy and infected grains and excitation spectra for 450 nm peak for healthy grain (**d**). The spectra corresponding to healthy and infected cases have green and red color, correspondingly.

Figure 7 shows the obtained luminescence spectra in the range 600–1000 nm with 400 nm excitation. The luminescence spectra of healthy grains (Figure 7a) show a band with a maximum at 674 nm and a shoulder on the long wavelength side at 718 nm. These peaks were attributed to chlorophyll α luminescence [52,53]. The excitation spectra of the 670 nm band are shown in Figure 7b. The luminescence excitation spectrum has a fairly wide band (400–550 nm) with a small maximum at 415 nm for a healthy sample, which is close to the Soret band in chlorophyll α. At the same time, for infected grains in the range of 600–1000 nm with the same excitation, the luminescence signal was an order of magnitude smaller, and no significant peaks were observed. The observation of the chlorophyll α peak in the red region (about 670 nm) allows one to consider them as a criterion for identifying healthy peaks.

When considering the luminescence spectra in the shorter wavelength range of 350–650 nm (Figure 7c) with 336 nm excitation wavelength, differences were also noted between the spectra for healthy and infected grains. First, a difference was noted in the wavelength for the luminescence maximum. Taking into account the healthy grains, the maximum was reached at 449 nm, which is close to the results reported for healthy grains of the winter wheat variety “Irishka No172” with 362 nm excitation [54].

It should be noted that the highest luminescence efficiency with the 450 nm peak excitation for healthy grains corresponds to a longer wavelength value, 372 nm; see Figure 7d. In order to obtain emission spectra in the 350–650 nm range for both healthy and infected samples and their differentiation, the 336 nm excitation is more convenient for express analysis than 372 nm.

The use of luminescent spectra in the range of 600–1000 nm with a 400 nm excitation looks more promising for practical needs due to several reasons. Firstly, a very intense luminescence of chlorophyll α, occurring in this range, is highly sensitive to the underlying processes in plants [55]. Secondly, the excitation wavelength and emission range are convenient for designing an optoelectronic device, for example, relatively cheap but sufficiently sensitive and high-quality silicon-based detectors, optimized optical filters and diffraction gratings, as well as laser excitation sources for visual ranges where the intensive chlorophyll α peak is situated [56,57]. Thirdly, the probability of luminescence excitation from possible impurities and pollutants is less. In addition, chlorophyll α luminescence has a more specific contour due to a relatively narrow peak around 670 nm and a shoulder around 720 nm.

#### 3.3.2. PCA of Luminescent Spectra

**Figure 8:**
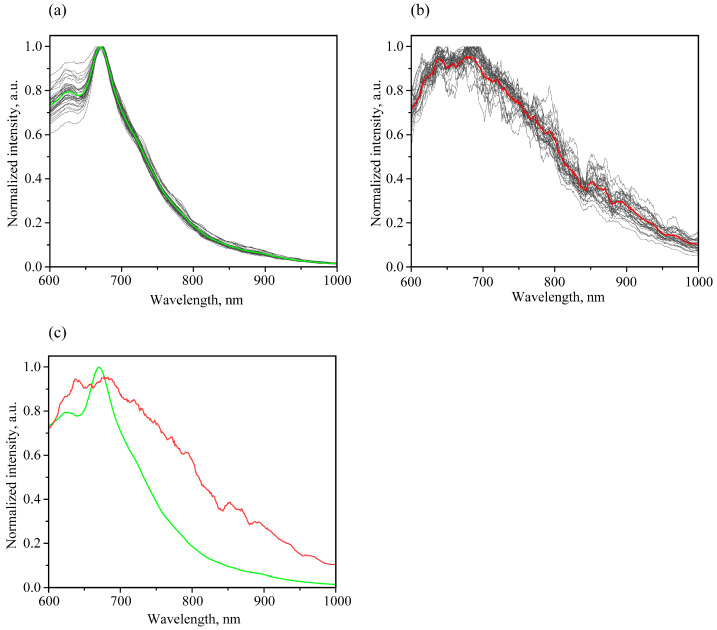
Normalized luminescence spectra of healthy samples (**a**), with green corresponding to the averaged spectra; infected samples (**b**), with red corresponding to the averaged spectra; and separate comparison of the averaged luminescence spectra of samples, where green is the case of healthy samples and red is the case of infected samples (**c**).

Based on the above reasons, it was decided to apply the PCA method for the analysis of normalized luminescence spectra in 600–1000 nm range with 400 nm excitation. It should be noted that the infected samples’ signal is much weaker than in the case of healthy ones, which leads to a significantly greater influence of possible noise on the intensity ratio in the luminescence spectrum, as well as greater signal variability when moving from grain to grain; see Figure 8a,b. However, on average, a characteristic feature for normalized spectra is a significantly higher relative intensity in the 700–1000 range nm for infected samples (Figure 8c).

**Figure 9:**
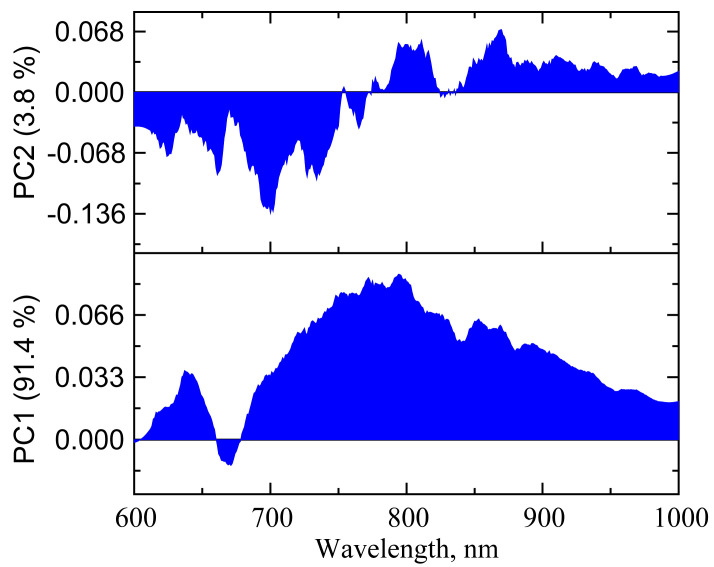
The loadings of first 2 PCs obtained during PCA analysis of luminescence data: bottom for PC1 and at the top for PC2.

**Figure 10:**
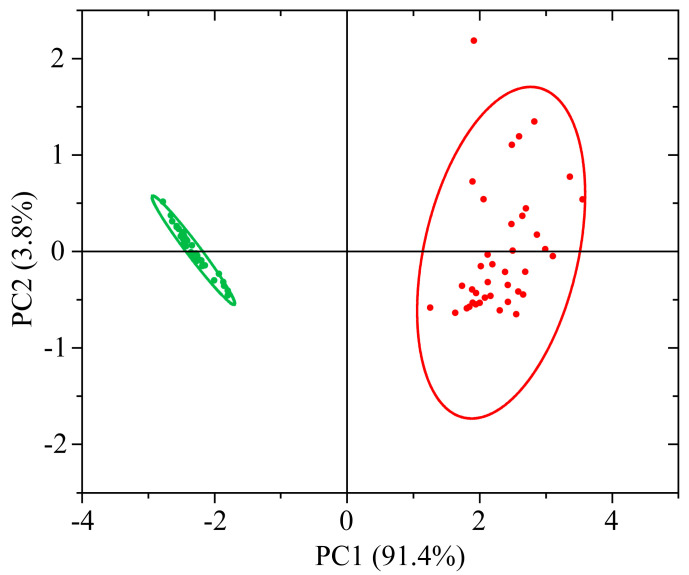
(PC1, PC2) combination of scores of principal components for healthy (green) and infected (red) samples with corresponding 95% probability ellipsoids.

This feature is manifested in the fact that for loadings of PC1, the most valuable are situated in this region (Figure 9). The maximum weight is about 800 nm for the PC1 loading plot. The PC1 makes it possible to explain 91.4% of the information. On the other hand, the next PC2 explains only 3.8%, and the other one less than 2% (Figure S5). In Figure 10, a fairly good separation for PC1 is noted. The Euclidean distance between the centers of ellipsoids of 95% probability is about 4.7 rel. units. At the same time, as can be seen from Figure 10, a rather small area of localization of values for healthy samples and a significantly larger area of localization for infected ones take place, which correlates with the data variation visually seen in Figure 8a,b.

#### 3.3.3. LDA and SVM Classification

Due to the strong localization of the two clusters, an unmistakable classification into healthy and infected grains was noted, even despite a fairly strong variation in the data for the infected sample, see Table 5. Mainly, a fairly strong difference in PC1 contributed to the correct separation. Thus, it can be concluded that automatic division of healthy and infected grains based on normalized spectra in the range of 600–1000 nm with 400 nm excitation can be used as an additional criterion for separating the grains. It should be noted that the use of these methods in combination is primarily due to the fact that the luminescence intensity for contaminated grains is very weak and rather broad, which can lead to fluctuations in the position of the maximum and the influence of noise after normalization. At the same time, for healthy grains, the observed luminescent peak from chlorophyll α is rather narrow and reproducible. Its strong luminescence leads to a significant reduction in the influence of possible differences in the baseline, as well as noise, which has a positive effect on the identification of healthy grains and can serve as an essential criterion if it is necessary to differentiate grains infected with ergot and healthy grains with a non-destructive method on an industrial scale.

## 4. Conclusions

The methods of optical spectroscopy were used to study the optical properties of healthy and ergot-infected grains of the winter wheat “Moskovskaya 56” cultivar infected with the fungi genus *Claviceps sp*. FTIR ATR absorption spectroscopy revealed the presence of a significant amount of protein media for infected samples. The PCA analysis demonstrated reliable classification with two first PCs. The loading of PC1 correlates with the difference spectrum and protein media. The maximum weights correlate with the most intense weights in lipids and fatty acids for the loading of PC2.

The addition of UV-vis-NIR reflectance spectroscopy allowed one to obtain not only vibrational data in the MIR region, but also give the information about overtones and combination vibrational modes, as well as information about electronic states, including such substances as pigments and metabolites. This approach allows the researcher to make a more effective scheme of classification on the basis of the set of spectral criteria that deal with chemical composition.

The study of the luminescent properties of healthy and infected grains showed the presence of an intense luminescence of chlorophyll α (peak at 670 nm and shoulder near 718 nm) with the effective 400 nm wavelength excitation for healthy samples. The presence of a wide luminescence band in the 700–900 nm range facilitates the separation of infected and healthy samples. This, in combination with a sufficiently strong chlorophyll α luminescence and a developed instrumentation base, has a number of prospects for further application in devices, in contrast to differentiation based on luminescence in the UV-visible range.

The spectral features for infected and healthy grains on the example of the winter wheat “Moskovskaya 56” cultivar were demonstrated. The use of PCA-LDA and PCA-SVM methods showed the possibility of accurate discrimination into healthy and infected grains at this infection stage with all three techniques.

In the future, this may contribute to the further development of diagnostic methods and the increasing implementation of machine learning systems for grain quality control in the framework of industrial streaming measurements, as well as in the analysis of large grain volumes.

## Figures and Tables

**Table 1:** Peak frequencies and their assignment in case of averaged spectrum of healthy samples.

Peak Frequency, cm^−1^	Assignment ^1^ on The Basis of [26,39,40,41,42,43]
893	stretching vibrations in glyosidic bonds
983	v(C-O)
1003	v(C-O)
1156	v_as_(COC)
1241	v(C-O)
1314	δ(HCC), δ(COH)
1365	δ(HCC), δ(COH)
1414	δ(XCH)
1453	δ(HCH)
1512	v(C=C), δ(XCH)
1598	v(C=C)
1629	δ(OHO)
1730	v(C=O)
2850	v_s_(CH_2_)
2918	v_as_(CH_2_)
3268	v(OH) in water and intra- and inter-molecular hydrogen bonding

^1^ The stretching and bending vibrations are denoted with v and δ symbols, respectively. The symmetric and antisymmetric vibrations are denoted with a and as symbols, respectively.

**Table 2:** Peak frequencies and their assignment in case of averaged spectrum of infected samples.

Peak Frequency, cm^−1^	Assignment ^1^ on The Basis of [26,39,40,41,42,43]
880	stretching vibrations in glyosidic bonds
1027	v(CO)
1151	v_as_(COC)
1233	amide III
1310	δ(HCC), δ(COH)
1378	δ(HCC), δ(COH)
1447	δ(HCH)
1534	amide II
1633	amide I, δ(OHO)
1738	v(C=O)
2850	v_s_(CH_2_)
2920	v_as_(CH_2_)
3056	v(=C-H)
3181	v(NH)
3266	v(OH) in water and intra- and inter-molecular hydrogen bonding

^1^ The stretching and bending vibrations are denoted with v and δ symbols, respectively. The symmetric and antisymmetric vibrations are denoted with a and as symbols, respectively.

**Table 3:** Evaluation of accuracy, sensitivity and specificity, positive predictive value and negative predictive value for FTIR spectra data.

Model	Scores Data	Data Set	Accuracy	Sensitivity	Specificity	Positive Predictive Value	Negative Predictive Value
PCA-LDA	PC1, PC2	Training Set	1.00	1.00	1.00	1.00	1.00
		Prediction Set	1.00	1.00	1.00	1.00	1.00
		Total Set	1.00	1.00	1.00	1.00	1.00
	PC1, PC3	Training Set	1.00	1.00	1.00	1.00	1.00
		Prediction Set	1.00	1.00	1.00	1.00	1.00
		Total Set	1.00	1.00	1.00	1.00	1.00
	PC2, PC3	Training Set	0.60	0.65	0.54	0.59	0.61
		Prediction Set	0.73	0.62	0.85	0.80	0.69
		Total Set	0.62	0.62	0.64	0.62	0.64
	PC1, PC2, PC3	Training Set	1.00	1.00	1.00	1.00	1.00
		Prediction Set	1.00	1.00	1.00	1.00	1.00
		Total Set	1.00	1.00	1.00	1.00	1.00
PCA-SVM	PC1, PC2	Training Set	1.00	1.00	1.00	1.00	1.00
		Prediction Set	1.00	1.00	1.00	1.00	1.00
		Total Set	1.00	1.00	1.00	1.00	1.00
	PC1, PC3	Training Set	1.00	1.00	1.00	1.00	1.00
		Prediction Set	1.00	1.00	1.00	1.00	1.00
		Total Set	1.00	1.00	1.00	1.00	1.00
	PC2, PC3	Training Set	0.83	0.77	0.88	0.87	0.79
		Prediction Set	0.92	0.85	1.00	1.00	0.87
		Total Set	0.86	0.79	0.92	0.91	0.82
	PC1, PC2, PC3	Training Set	1.00	1.00	1.00	1.00	1.00
		Prediction Set	1.00	1.00	1.00	1.00	1.00
		Total Set	1.00	1.00	1.00	1.00	1.00

**Table 4:** Evaluation of accuracy, sensitivity and specificity, positive predictive value and negative predictive value for UV-vis-NIR spectra data.

Model	Score Data	Data Set	Accuracy	Sensitivity	Specificity	Positive Predicted Value	Negative Predicted Value
PCA-LDA	PC1, PC2	Training Set	1.00	1.00	1.00	1.00	1.00
		Prediction Set	1.00	1.00	1.00	1.00	1.00
		Total Set	1.00	1.00	1.00	1.00	1.00
	PC1, PC3	Training Set	1.00	1.00	1.00	1.00	1.00
		Prediction Set	1.00	1.00	1.00	1.00	1.00
		Total Set	1.00	1.00	1.00	1.00	1.00
	PC2, PC3	Training Set	0.54	0.43	0.64	0.55	0.53
		Prediction Set	0.43	0.43	0.43	0.43	0.43
		Total Set	0.50	0.43	0.57	0.50	0.50
	PC1, PC2, PC3	Training Set	1.00	1.00	1.00	1.00	1.00
		Prediction Set	1.00	1.00	1.00	1.00	1.00
		Total Set	1.00	1.00	1.00	1.00	1.00
PCA-SVM	PC1, PC2	Training Set	1.00	1.00	1.00	1.00	1.00
		Prediction Set	1.00	1.00	1.00	1.00	1.00
		Total Set	1.00	1.00	1.00	1.00	1.00
	PC1, PC3	Training Set	1.00	1.00	1.00	1.00	1.00
		Prediction Set	1.00	1.00	1.00	1.00	1.00
		Total Set	1.00	1.00	1.00	1.00	1.00
	PC2, PC3	Training Set	0.96	1.00	0.93	0.93	1.00
		Prediction Set	0.93	1.00	0.86	0.88	1.00
		Total Set	0.95	1.00	0.90	0.91	1.00
	PC1, PC2, PC3	Training Set	1.00	1.00	1.00	1.00	1.00
		Prediction Set	1.00	1.00	1.00	1.00	1.00
		Total Set	1.00	1.00	1.00	1.00	1.00

**Table 5:** Evaluation of accuracy, sensitivity and specificity, positive predictive value and negative predictive value for luminescence spectra data.

Model	Scores Data	Data Set	Accuracy	Sensitivity	Specificity	Positive Predicted Value	Negative Predicted Value
PCA-LDA	PC1, PC2	Training Set	1.00	1.00	1.00	1.00	1.00
		Prediction Set	1.00	1.00	1.00	1.00	1.00
		Total Set	1.00	1.00	1.00	1.00	1.00
PCA-SVM	PC1, PC2	Training Set	1.00	1.00	1.00	1.00	1.00
		Prediction Set	1.00	1.00	1.00	1.00	1.00
		Total Set	1.00	1.00	1.00	1.00	1.00

## Data Availability

The data of the current study are available from the corresponding authors on reasonable request.

## Supplementary Materials

The following supporting information can be downloaded at: https://www.mdpi.com/article/10.3390/foods12183426/s1, Figure S1: Dependence of the percentage of variance on the number of the main component (red), dependence of the total explained percentage of information depending on the number of the main component (blue) for the FTIR absorbance data; Figure S2: Combination of scores of principal components: a—(PC1, PC2, PC3), b—(PC1, PC2), c—(PC1, PC3), d—(PC2, PC3) for the FTIR absorbance data for healthy (green) and infected (red) samples with the corresponding 95% probability ellipsoids; Figure S3: Dependence of the percentage of variance on the number of the main component (red), dependence of the total explained percentage of information depending on the number of the main component (blue) for the UV-vis-NIR absorbance data, Figure S4: Combination of scores of principal components: a—(PC1, PC2, PC3), b—(PC1, PC2), c—(PC1, PC3), d—(PC2, PC3) for the UV-vis-NIR absorbance data for healthy (green) and infected (red) samples with the corresponding 95% probability ellipsoids; Figure S5: Dependence of the percentage of variance on the number of the main component (red); dependence of the total explained percentage of information depending on the number of the main component (blue) for the luminescence data.
