# Supplementary material for: Investigation of Spectroscopic Peculiarities of Ergot-Infected Winter Wheat Grains
Source: Foods. 2023 Sep 14;12(18):3426. doi: 10.3390/foods12183426 (PMC10528831; doi:10.3390/foods12183426)
Supplement: Supplementary file 1 [file foods-12-03426-s001.zip › foods-2468534-supplementary.pdf]

Supporting information

Investigation of Spectroscopic Peculiarities of Ergot-Infected Winter Wheat Grains

Dmitrii Pankin[1], Anastasia Povolotckaia[1*], Eugene Borisov[1], Alexey Povolotskiy[2], Sergey Borzenko[3], Anatoly Gulyaev[3], Stanislav Gerasimenko[3], Alexey Dorochov[3], Viktor Khamuev [3], Maksim Moskovskiy[3]

[1] Center for Optical and Laser Materials Research, St. Petersburg State University, Ulianovskaya 5, 198504 St. Petersburg, Russia; dmitrii.pankin@spbu.ru (D.P.); anastasia.povolotckaia@spbu.ru (A.P.); eugene.borisov@spbu.ru (E.B.)

[2] Institute of Chemistry, St. Petersburg State University, Universitetskii pr. 26, 198504 St. Petersburg, Russia; alexey.povolotskiy@spbu.ru (A.P.)

[3] Federal Scientific Agro-Engineering Center VIM, 1st Institutskiy proezd 5, 109428 Moscow, Russia; serzhbk@gmail.com (S.B.); tomasss1086@mail.ru (A.G.); stanislav.mkm@gmail.com (S.G.); dorokhov@rgau-msha.ru (A.D.); viktor250476@yandex.ru (V.H.); maxmoskovsky74@yandex.ru (M.M.)

*Correspondence: anastasia.povolotckaia@spbu.ru

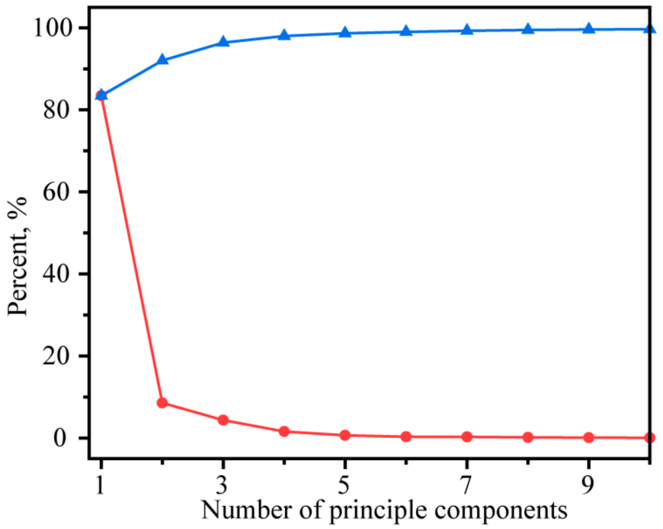

Figure S1. Dependence of the percentage of variance on the number of the main component (red), dependence of the total explained percentage of information depending on the number of the main component (blue) for the FTIR absorbance data.

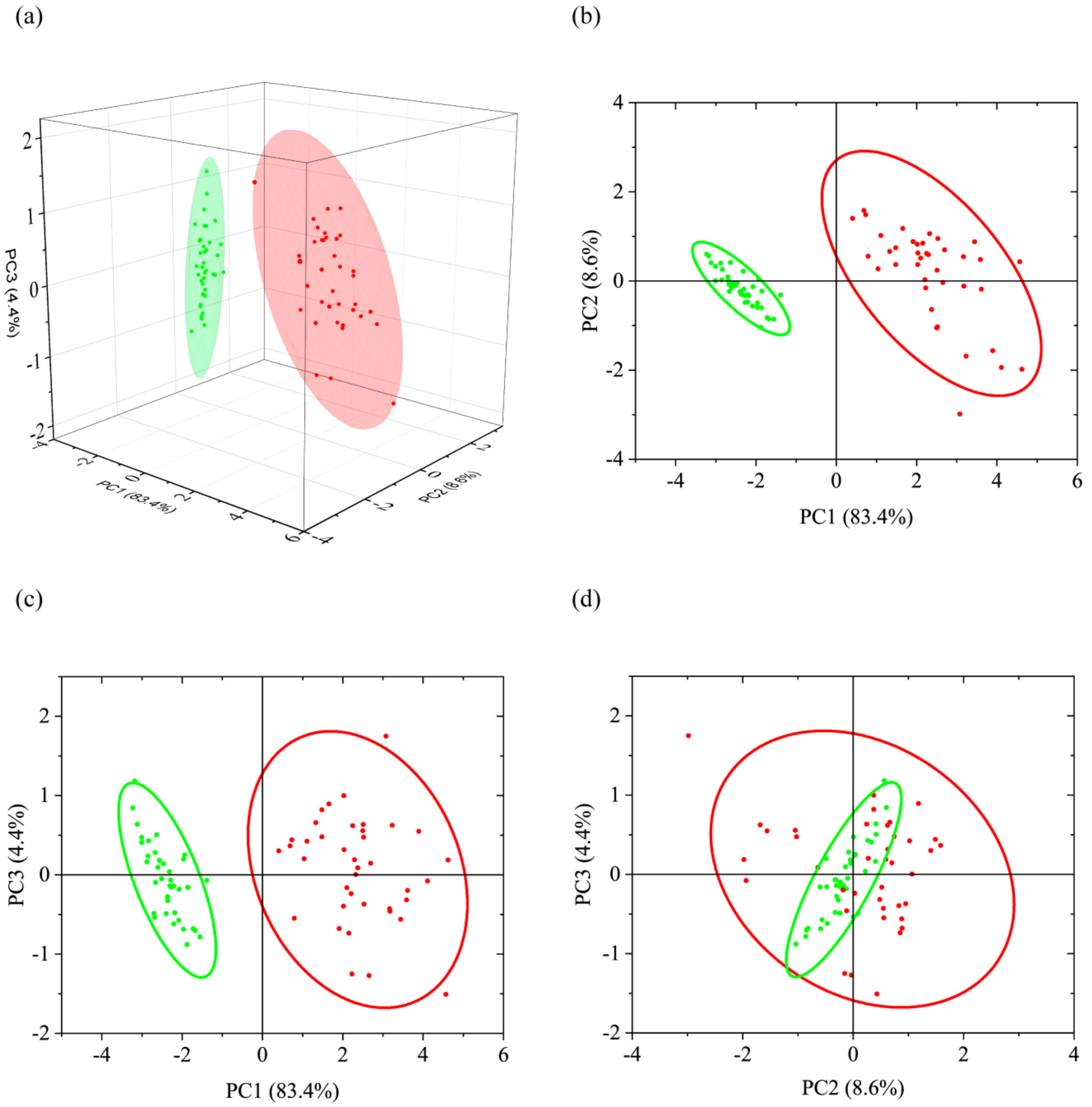

Figure S2. Combination of scores of principal components: a – (PC1,PC2,PC3), b – (PC1,PC2), c – (PC1,PC3), d – (PC2,PC3) for the FTIR absorbance data for healthy (green) and infected (red) samples with the corresponding 95% probability ellipsoids.

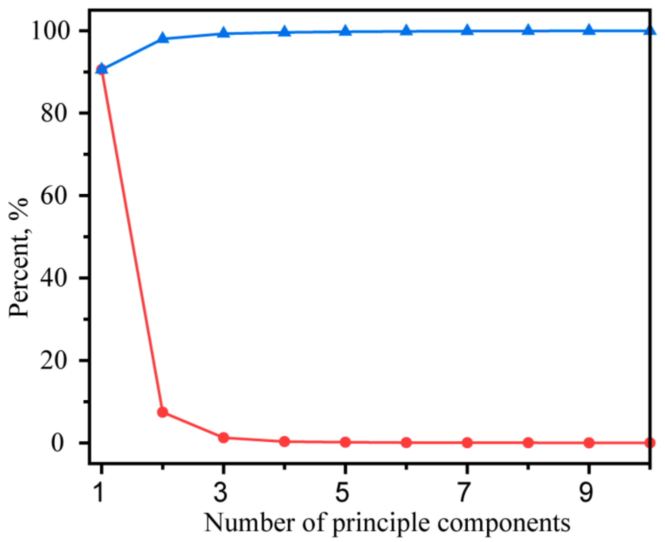

Figure S3. Dependence of the percentage of variance on the number of the main component (red), dependence of the total explained percentage of information depending on the number of the main component (blue) for the UV-vis-NIR absorbance data.

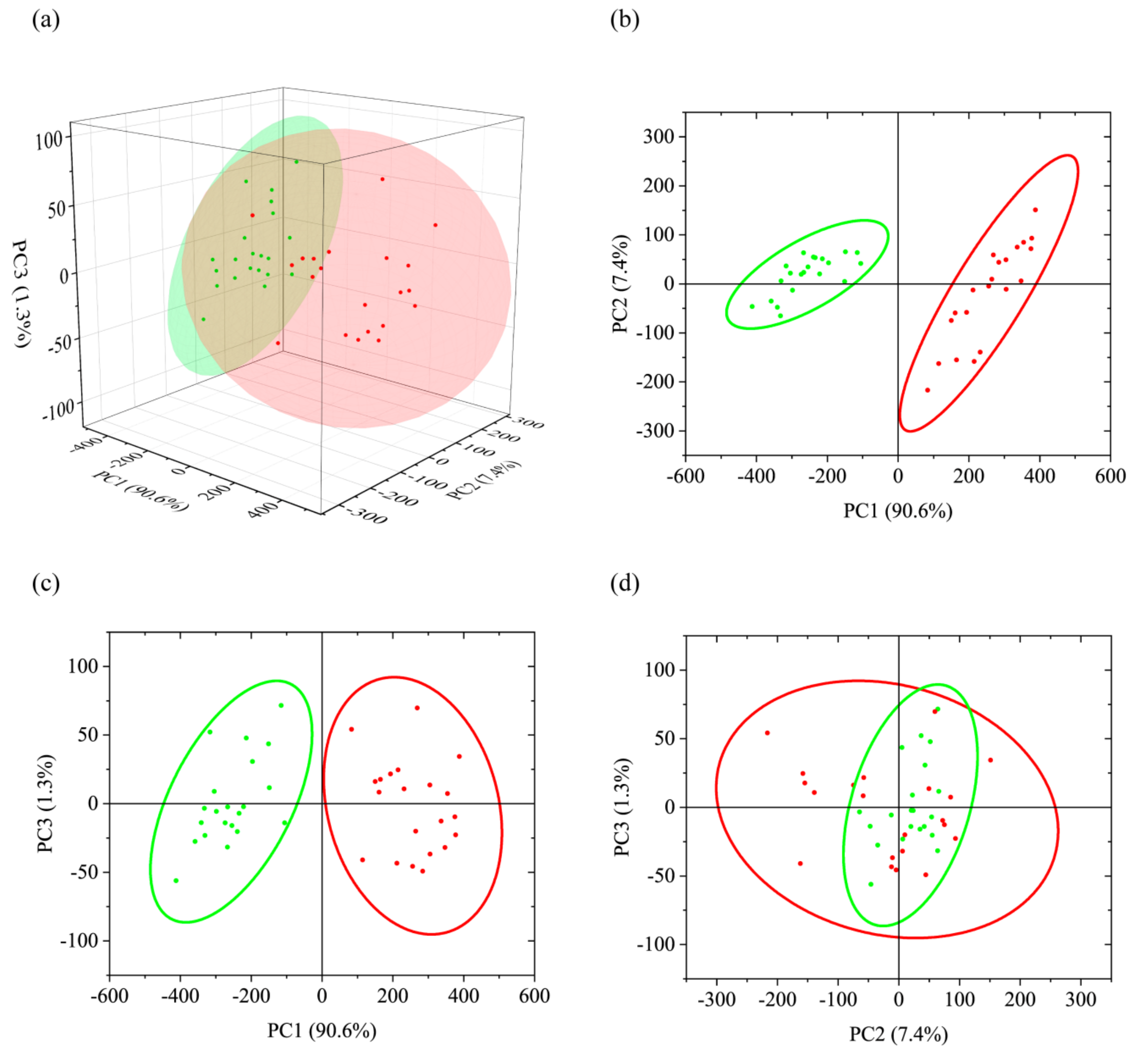

Figure S4. Combination of scores of principal components: a – (PC1,PC2,PC3), b – (PC1,PC2), c – (PC1,PC3), d – (PC2,PC3) for the UV-vis-NIR absorbance data for healthy (green) and infected (red) samples with the corresponding 95% probability ellipsoids.

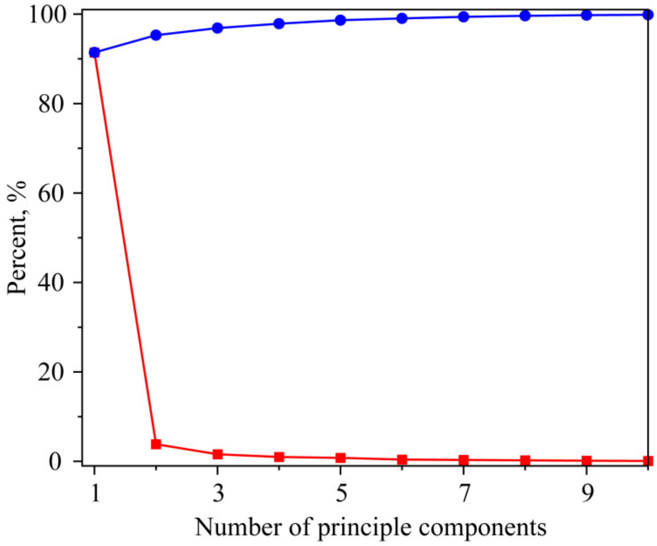

Figure S5. Dependence of the percentage of variance on the number of the main component (red), dependence of the total explained percentage of information depending on the number of the main component (blue) for the luminescence data.
